# Rock Reservoir Properties from the Comprehensive Interpretation of Nuclear Magnetic Resonance and Mercury Injection Porosimetry Laboratory Results

**DOI:** 10.1007/s00723-014-0617-4

**Published:** 2014-11-28

**Authors:** Jadwiga A. Jarzyna, Paulina I. Krakowska, Edyta Puskarczyk, Roman Semyrka

**Affiliations:** Faculty of Geology, Geophysics and Environment Protection, AGH University of Science and Technology, al. Mickiewicza 30, 30-059 Krakow, Poland

## Abstract

Combination of laboratory measurements res ults of various properties, i.e. porosity, density, permeability and mineral composition, was done to get additional factors useful in fluid flow description in the Miocene sandy-shaly formation. Special computer processing of nuclear magnetic resonance outcomes and mercury injection porosimetry results turned out to be useful in the estimation of the relationships facilitating the reservoir characterization and defining new helpful factors. Determination of the relationships between groups of quantities describing pore space of rock formation was presented as the basis for permeability prediction and for relationships extrapolation into interesting areas.

## Introduction

Reservoir clastic rocks, investigated from the very beginning of petrophysics as a branch of the Earth sciences, now need specific and more sophisticated approach to obtain parameters for calculating reserves of hydrocarbons and waters and also determining ability to exploitation [1–4]. History of Polish works aimed to recognize the Miocene reservoir properties is long. There is plenty of works classified as geological studies [5–7] and well-logging interpretation [8–10] and seismic inversion examples [11–13] and comprehensive petrophysical analysis [14–17]. Many of researchers used the results of laboratory experiments which were carried out on available equipment on rock plugs. The presented work belongs to the last group and illustrates one more effort towards developing the analyses of rocks reservoir properties.

The Miocene formation at the Carpathian Foredeep is a rewarding material for petrophysical investigations. Sandstone-mudstone-claystone thinly-bedded mix of great instability of properties due to lithological changes exemplifies a challenge for petrophysicists working with conventional methods of rock properties determination. On the other side it makes a link between difficult conventional and unconventional reservoir rocks. Methods used in the petrophysical properties investigations and analysis in the Miocene formation provided the relationships which can be extended to properties ranges characteristic for unconventional reservoirs. Detailed analyses drew also attention to necessity of individual treatment of various parts of the formation.

One of the goals of the petrophysical analysis was to build relationships between parameters and factors determined from rock sample laboratory investigations facilitating determination of one group of parameters on the basis of the other. Attention was also focused on the determination of factors characterizing pore space which are difficult to be parameterized. Ability of fluid flow determination in the porous Miocene sandstone-mudstone-claystone formation was crucial among other aspects important in reservoir characterization.

Methods of the laboratory investigations on core plugs used in the presented analysis [helium pycnometer (HP), mercury injection porosimetry (MIP) and nuclear magnetic resonance (NMR)] are known from literature and practice. Many research groups confirmed their usefulness in petrophysical analyses oriented to determine the reservoir properties [18–24]. In the paper the specific approach to MIP outcomes resulted from division of the pressure vs. mercury volume curve into three parts related to Thomeer hyperbolas fitted to different pore systems was used. Next, similar approach was extended into NMR results and emphasis was placed to combining the parameters from various methods.

## Data Sets

**Fig. 1:**
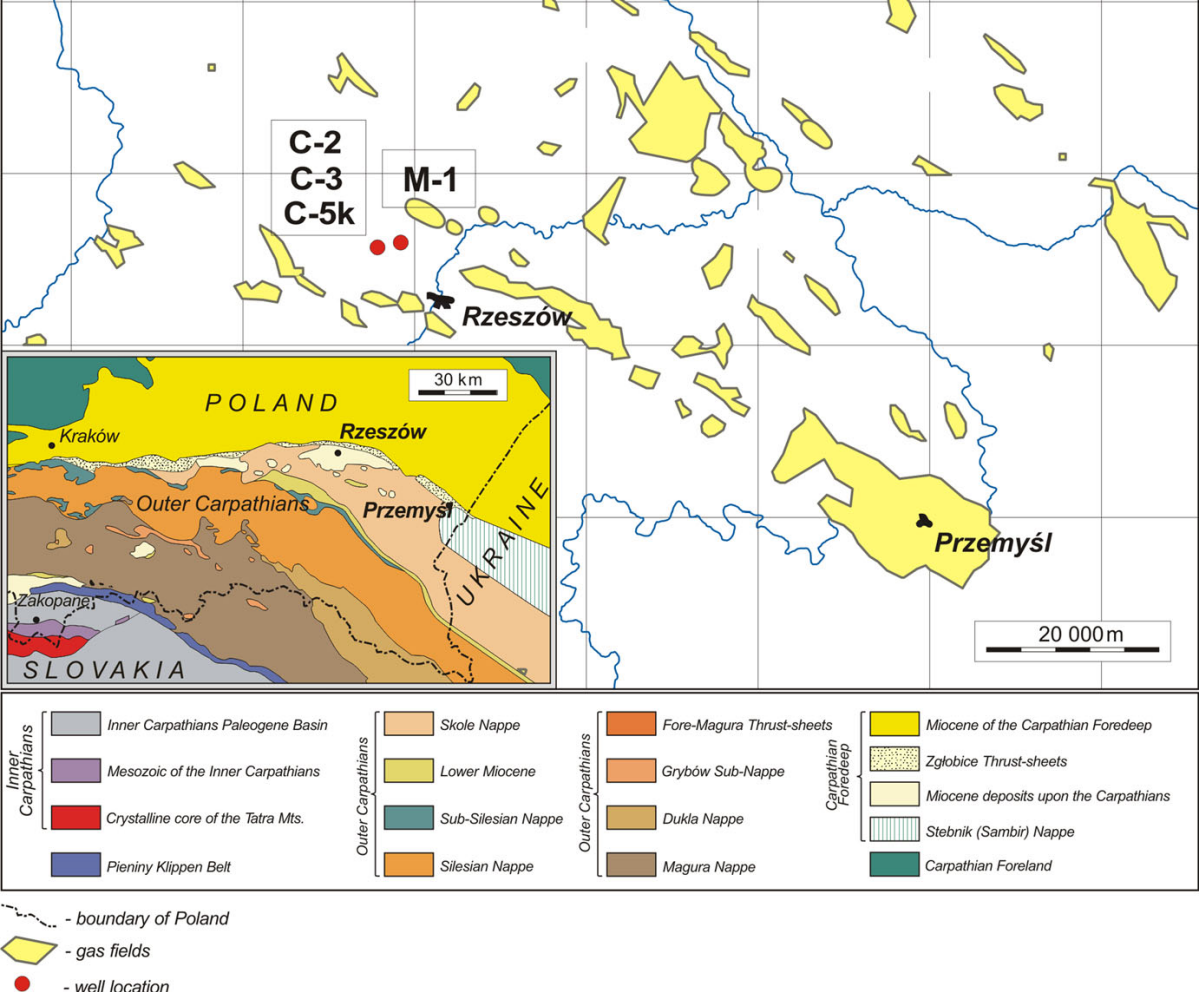
Location of wells (C-2, C-3, C-5k, M-1) from which cores for laboratory measurements were obtained [6, 25, 26]

**Fig. 2:**
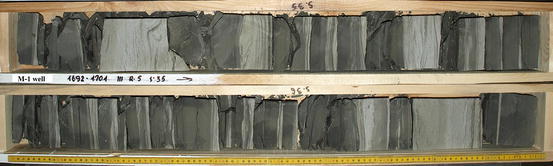
Cores from M-1 well in the depth section 1,692–1,701 m; Sarmatian sandstone-mudstone-claystone thinly-bedded formation, case nr 3 (photo S. J. Porebski)

Authors had to their disposal laboratory experiments results of 52 rock samples from 4 wells (M-1–33 samples, C-2–5, C-3–4 and C-5k–10), all located in the Carpathian Foredeep (Fig. 1). Only Sarmatian thinly-bedded sandstone-mudstone-claystone formation was investigated (Fig. 2). Depths of cores covered the range of 1,254.10–1,739.10 m. Rocks at the selected depth sections were built as packets formed by sandstones, claystones and mudstones laminas in various proportions. Packets were of a few dozen meters in thickness, larger in the upper part and smaller in the lower part. Deltaic siliciclastic rocks were primarily deposited in a low energy environment and next exposed to the pseudo tectonic local activity partially related to the movement of the Carpathian orogene to the north.

Standard size cylindrical core plugs (radius 1″ and height 1.14″) were used for laboratory investigations. Intention of authors was to make all laboratory analyses on the same pieces of rocks and in the majority of cases it was fulfilled. Destructive mercury injection porosimetry (MIP) measurements were done as the last. Sarmatian sandstone- mudstone-claystone formation in situ drilled by the wells was only saturated with formation water, so for the laboratory experiments core plugs were filled with brine of 30 g/l mineralization, typical for the region of investigation.

Laboratory investigations provided the following rock properties: bulk density (*δ*_hp_), total porosity (*Φ*_hp_), absolute permeability (*K*), natural radioactivity (volume of potassium, uranium and thorium) and mineral composition from roentgen analysis (XRD). NMR measurements provided the total porosity (Kp nmr), dynamic porosity or Free Fluid Index (FFI) and irreducible water saturation (Swirr). Available complete digital NMR outcomes enabled to construct *T*_2_ distributions and cumulative distributions of NMR signals. MIP was the source of specific parameters related to pores structure and size, i.e. total intrusion volume (Bv all), total pore area, median pore diameter (*D* *m*) calculated on the basis of pore volume, bulk density (*δ*_mp_) and porosity (*Φ*ef mp). Available complete porosimetry raw data enabled preparing standard plots illustrating measured parameters as: cumulative volume of mercury (*S* Hg, incremental volume of mercury (*S* Hg inc), cumulative pore area (*A*) and incremental pore area (*A* inc) as functions of pore diameter (*D*). Digital raw quantities worked also as input data into software used to calculate Thomeer hyperbolas and Swanson parameters. Selected parameters of several typical samples are presented in Table 1.

**Table 1:** Selected parameters of several typical Miocene rock samples (explanations in text and at the end of paper)

No. of sample	*δ*_hp_ [g/cm^3^]	*Φ*_hp_ [%]	*K* [mD]	*Φ*ef mp [frac.]	Bv all [frac.]	Kp nmr [frac.]	Kp nmr *ef* [frac.]	Swirr [%]	*D* [mm]
9,811	2.11	21.59	29.19	0.2090	0.1077	22.69	15.92	46.01	0.3700
9,812	2.13	21.88	83.37	0.1601	0.0788	21.10	15.73	38.39	0.1452
9,817	2.01	25.45	198.40	0.0728	0.0267	26.71	20.76	32.95	0.3176
9,818	2.29	15.90	0.29	0.1526	0.6780	20.62	6.70	87.00	0.0535
9,822	2.07	26.30	976.89	0.1484	0.7210	22.19	20.12	14.56	0.1204
9,828	2.15	20.77	21.98	0.0700	0.0414	23.02	13.61	56.55	0.0723
9,831	2.38	12.76	0.01	0.1375	0.0186	14.08	3.93	85.23	0.0705
9,055	2.16	19.65	19.07	0.1193	0.0612	21.06	11.89	43.54	0.0612

Values in Table 1 illustrated the properties variability of the Miocene rock formation. Comparison of porosity determined using various laboratory methods presented influence of different physical basis on the obtained results

## Mercury Injection Porosimetry (MIP) Results

**Fig. 3:**
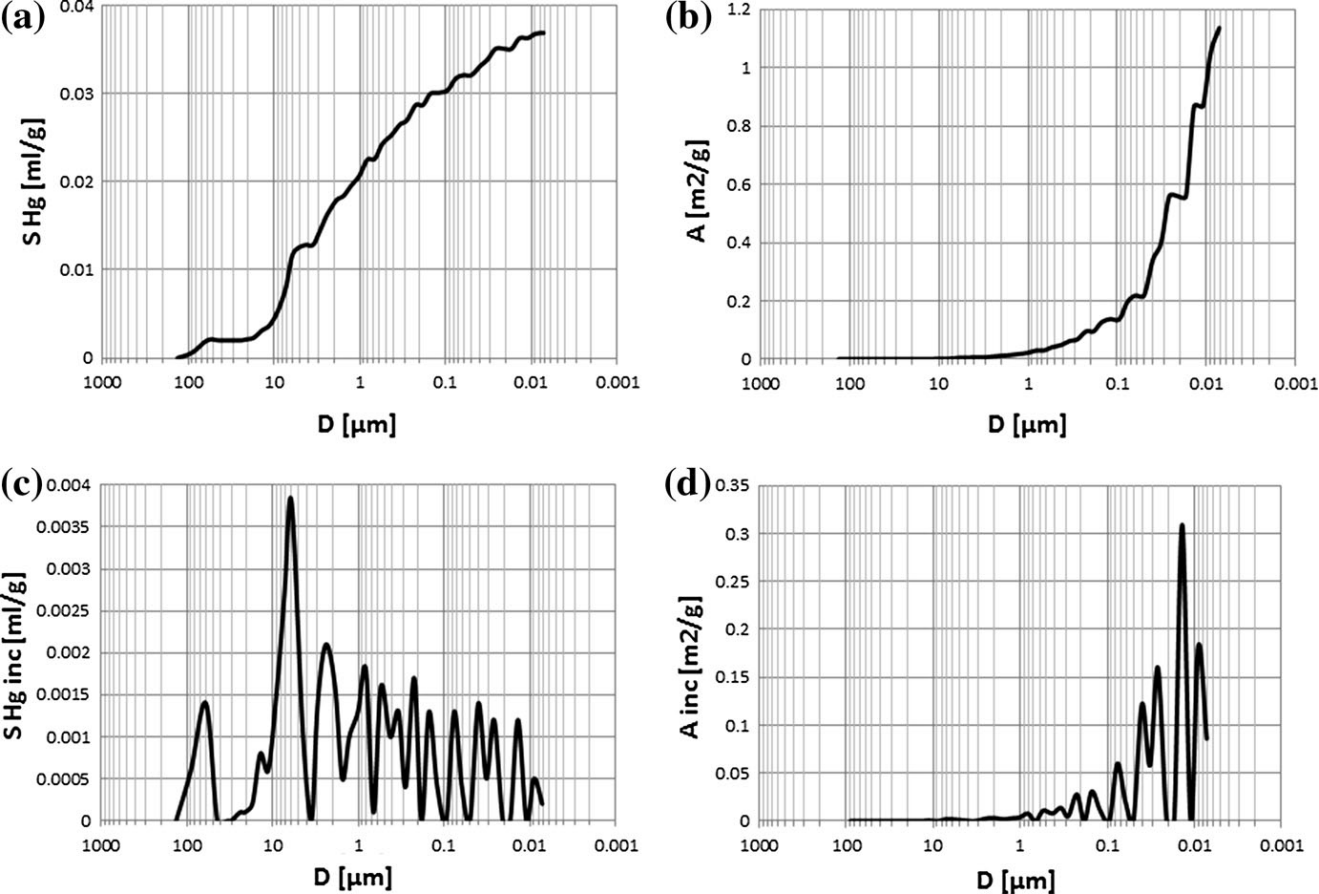
**a** Cumulative mercury volume, *S* Hg, vs. pore diameter, *D*, sample 9,814, porous-fractured system. **b** Cumulative pore area, *A*, vs. pore diameter, *D*, sample 9,814, porous-fractured system. **c** Incremental mercury volume, *S* Hg inc, vs. pore diameter, *D*, sample 9,814, porous-fractured system. **d** Incremental pore area, *A* inc, vs. pore diameter, *D*, sample 9,814, porous-fractured system

**Fig. 4:**
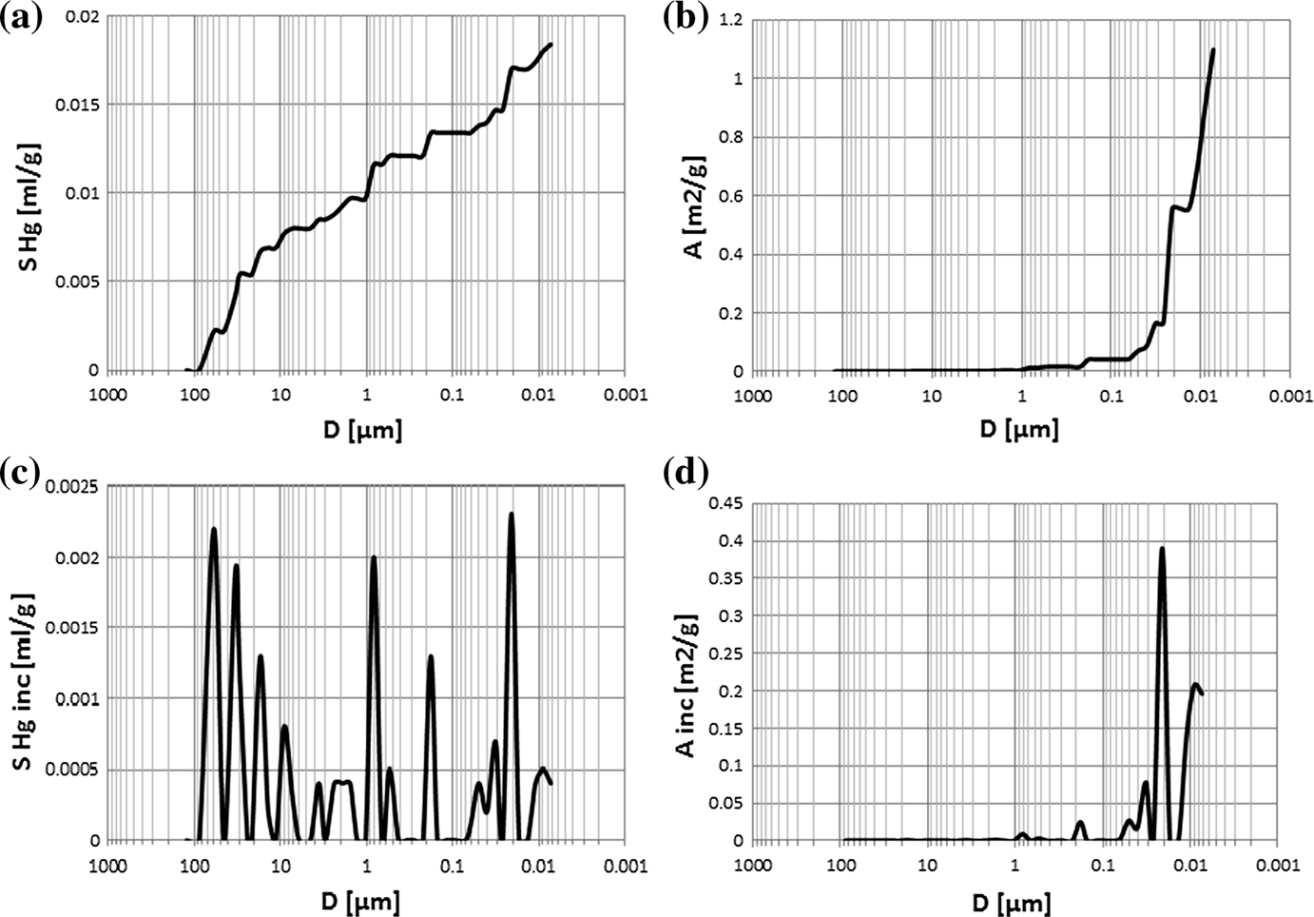
**a** Cumulative mercury volume, *S* Hg, vs. pore diameter, *D*, sample 9,068, fractured system. **b** Cumulative pore area, *A*, vs. pore diameter, *D*, sample 9,068, fractured system. **c** Incremental mercury volume, *S* Hg inc, vs. pore diameter, *D*, sample 9,068, fractured system. **d** Incremental pore area, *A* inc, vs. pore diameter, *D*, sample 9,068, fractured system

Mercury injection porosimetry (MIP) is the useful tool for investigating the pore-level heterogeneity [24]. Standard raw outcomes provided four plots enabling quick look analysis and primary recognition of pore system type in the investigated rocks (Figs. 3, 4). In the discussed case the majority of rock samples revealed porous-fractured systems. Some of them showed only fractured pore system (Fig. 4). At the first section of plots (pore radii of 150–10 μm) the boundary anomaly (boundary effect) related to pores opened during the plug preparation was visible [27]. Authors assumed that this anomaly was not the representative of mercury volume which took part in the mercury injection and removed it from the next step of data processing and evaluating of pore systems. In the next section (10–1 μm) generally porous system was observed. Fractures were mainly identified in the last part of the plot. Porosimetry experiments were done using AutoPore II 9220 porosimeter (Micrometrics Co.). Maximal injection pressure was 60,000 psi (413.3 MPa), while minimal–lower than ambient pressure. Working fluid, satisfying the condition of non-wetting liquid, was mercury. Sample was placed in a vessel which is filled with mercury under vacuum conditions. Then the pressure was gradually increased causing mercury injection to the porous space of the rock sample.

**Fig. 5:**
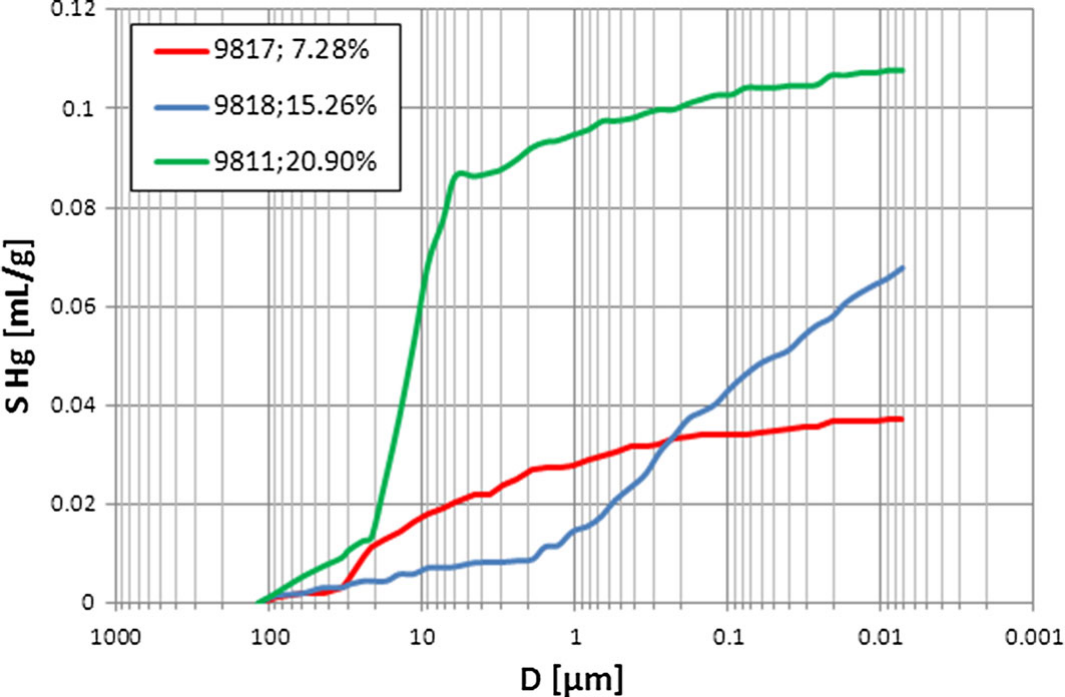
Cumulative volume of mercury, *S* Hg, vs. pore diameter, *D*; curve parameter–*Φ*ef mp

**Fig. 6:**
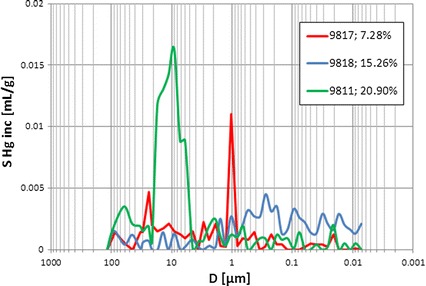
Incremental volume of mercury, *S* Hg inc, vs. pore diameter, *D;* curve parameter–*Φ*ef mp

Collective presentation of cumulative volume of mercury vs. pore diameter plots showed differences in porous-fractured systems (Fig. 5). Curve related to sample 9817 (*Φ*ef mp = 7.28 %) revealed constant gradient. It meant that pores of different diameters took part in mercury intrusion in similar proportion. In this sample one pore system was identified. Curve related to sample 9811 (*Φ*ef mp = 20.90 %) had two pore systems distinctly visible. Collective presentation of the cumulative (Fig. 5) and incremental (Fig. 6) volume of mercury vs. pore diameter supplemented the qualitative interpretation. The shape of plots showed differences in groups of pores engaged into mercury intrusion. Majority of examined samples showed concave plots but sample 9,818 (*Φ*ef mp = 15.26 %) revealed convex shape. In this case small part of intruded mercury volume occupied large pores and in the section of plot related to small pore diameters distinct increase of mercury volume was observed. Two pore systems were distinguished in this sample.

Cumulative volume of intruded mercury reflected effective porosity of rock sample. The extreme values were observed for the samples 9,811 and 9,817 (*Φ*ef mp = 20.90 and 7.28 %, respectively). The following regularity was observed at the pore diameter coordinate equal to 0.01 μm: the higher the effective porosity the higher the volume of intruded mercury was observed (Fig. 5). In the larger pore diameter range cumulative mercury volume routes had variable courses (Figs. 4, 5). One of the reasons is presence of fractures (Fig. 4).

**Fig. 7:**
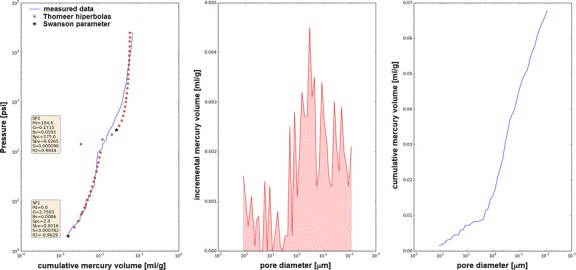
Results of semiautomatic processing of the MIP data using specific software [28]; sample 9,818, *Φ*ef mp = 15.26 %

Primary analysis of the MIP results was easily made using semiautomatic processing of the raw data with special software [28]. Raw outcomes were processed and three plots were graphically presented (Fig. 7) and parameters of Thomeer hyperbolas and Swanson coordinates were calculated [29–32]. Computerized processing of cumulative volume of mercury vs. pressure from MIP curves provided semiautomatic division into 1, 2 or 3 pore systems and Swanson parameter coordinates. Next, the results were used for constructing the relationships between reservoir parameters: effective porosity, permeability and other factors from NMR and Swanson parameter.

Each pore system (SP1, SP2, SP3) identified in the computerized processing was characterized by several quantities: Thomeer hiperbola may be presented in pore pressure (Pc) [psi] vs. bulk volume occupied by mercury (Bv) [ml/g] coordinate frame (Fig. 7), Swanson parameter, *S* (coordinates: Spc vs. Sbv), threshold pressure or extrapolated displacement pressure (Pd_*i*_) and effective porosity of system (Bv_*i*_). Swanson parameter coordinates were defined as bulk volume of mercury (Bv*)* and pore pressure (*P*c*)* at the inflection point on the Pc vs. Bv plot. Threshold pressure (Pd_*i*_) and effective porosity (Bv_*i*_) of selected pore systems mean pressure at which mercury starts to intrude into the pore system and bulk volume occupied by mercury in that pore system, respectively.

Plots like in Fig. 7 were the basis for determining the pore systems in rock sample (SP1, SP2 and SP3). Thomeer hyperbolas and Swanson parameters were automatically calculated for each pore system.

**Fig. 8:**
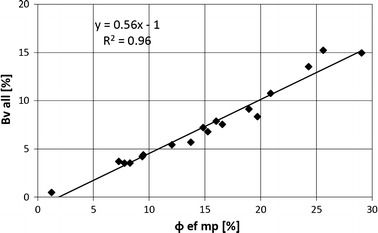
Comparison of effective porosity calculated as a sum of mercury volume injected in the determined pore systems, Bv all, vs. effective porosity from porosimetry, *Φ*ef mp

Dispersion plot of Bv all vs. porosity from standard porosimetry measurements, *Φ*_hp_, showed distinctly the difference in the porosity values (Fig. 8). Bv all means sum of MIP effective porosity in all selected systems. The level of the difference was up to 14.95 %. Difference was well correlated with Bv all and also with porosity from MIP. The discussed results confirmed the assumption that there was a systematic lowering of Bv all related to boundary effect. Small difference was observed for low-porosity samples, high one was characteristic for high-porosity samples. This regularity confirmed relationship between boundary effect (higher for high porosity samples) and difference in porosity from MIP and HP.

**Fig. 9:**
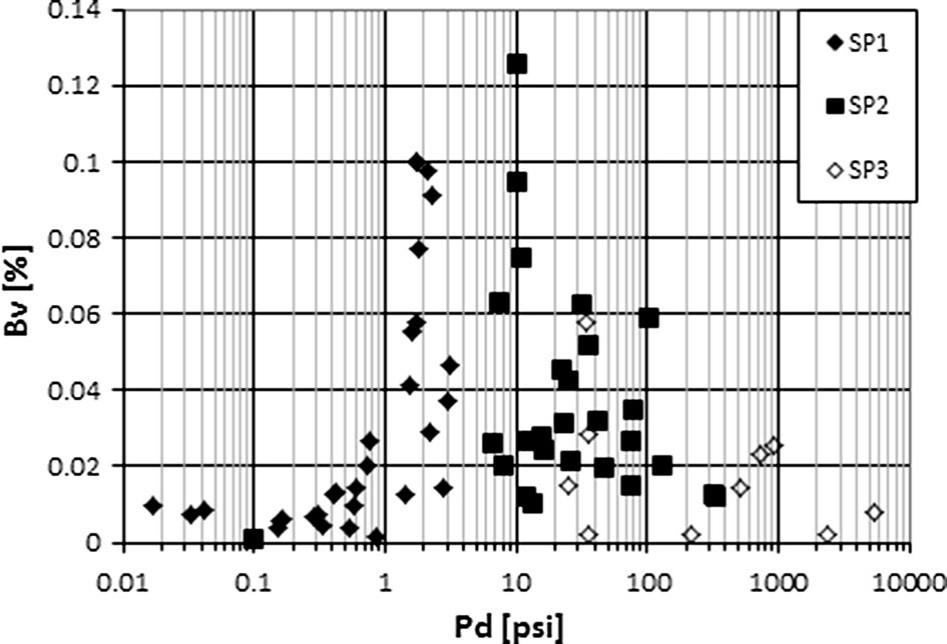
Selection of three pore systems (SP1, SP2, SP3) in rock sample on the basis of bulk volume of mercury in the system (Bv_*i*_) and pressure (Pd_*i*_)

Porosimetry results enabled separation of three or two or one pore systems in each rock sample on the basis of Thomeer hyperbolas construction. Boundary pressure between pore systems increased for systems SP1, SP2 and SP3. So, system SP1 was identified in the part of plane (Pc_*i*_ vs. Bv_*i*_ *, i* = *1,2,3*) of the smallest pressures and smallest intruded mercury volumes (Fig. 7). That part of Pc_*i*_ vs. Bv_*i*_ plane corresponded to the part of Bv_*i*_ vs. *D*_*i*_ plane with the smallest intruded mercury volume and largest pore diameters. Division into three pore systems (SP1, SP2 and SP3) was also observed in the plane Bv_*i*_ vs. Pd_*i*_ and *Φ*ef mp vs. Pd_*i*_ (Fig. 9). Bv_*i*_ and Pd_*i*_ were defined as bulk volume occupied by mercury in selected pore system and pressure at which mercury started to intrude into selected pore system, respectively.

## Nuclear Magnetic Resonance Laboratory Results

Proton-free precession is a physical basis of NMR experiment which delivers information nearly exclusively about hydrogen in rock formation. Hydrogen in rock occurs as water or hydrocarbons in pores, bound water in clay minerals and included in hydroxyl groups in these minerals and chemically bound water. NMR advantage is unique ability to distinguish dynamic porosity comprising only media in pores which can be moved from reservoir and media which will stay in reservoir. In other words, NMR precisely shows moveable water (free fluid index, FFI) and water bound in clay minerals and closed in pores by capillary forces. The basic parameters recorded in NMR experiment are: proton signal amplitude, spin–lattice relaxation time *T*_1_ and spin–spin relaxation time *T*_2_. These parameters are the source of detailed information about porosity (total and effective), irreducible water saturation and permeability of reservoir rocks, pore space structure and type of liquid filling rock pores. NMR measurements are based on Carr-Purcell-Meiboom-Gill pulse sequences recordings. CPMG sequence produces a closely-spaced series of spin echoes which are processed to determine transverse relaxation time *T*_2_ distributions [33, 34]. Three processes (bulk relaxation, surface relaxation and diffusion) influence hydrogen nuclei relaxation. In the multiphase systems, i.e. rocks with various media filling the pore space, the spin–spin relaxation curve can be presented as a sum of components (exponents), characterized by the relaxation time *T*_2*i*_. The continuous distribution of relaxation times is directly related to distribution of pore sizes in the rock sample examined. Exponential functions are used to describe time dependence of the signals from these three processes [35]. Exponent of the amplitude weighted NMR signal, labelled as *T*_2ML_ (Eq. 1) was applied as the useful factor in the comprehensive interpretation directed to combine results of the MIP outcomes and NMR results. The similar role served the natural logarithm of time-weighted average amplitude (Eq. 2) being a factor depending on porosity of sample.

1 $$T_{{ 2 {\text{ML}}}} = \exp \left(\frac{{\sum\limits_{i} {AS_{i} \cdot \ln T_{2i} } }}{{\sum\limits_{i} {AS_{i} } }}\right),$$

2 $${\text{Av}}{\_}{\text{amp}} = \frac{{\sum\limits_{i} {AS_{i} \cdot \ln T_{2i} } }}{{\sum\limits_{i} {\ln T_{2i} } }},$$

where: *T*_2ML_ *–*NMR mean logarithmic *T*_2_, AS_*i*_ *–*NMR signal amplitude normalized to maximal cumulative amplitude, Av_amp–NMR average amplitude.

NMR experiments were done in Oil and Gas Institute, Krakow, Poland using Maran-7 spectrometer equipped with permanent magnet producing the magnetic field of 0.187 T. Hydrogen nuclei precession frequency in that magnetic field is equal to 7.9 MHz. Investigations were made on plugs of length equal to 0.04 m and diameter equal to 0.0254 m. In such plugs it can be assumed that the stable magnetic field is homogeneous. All plugs were saturated with 30 g/l brine. Measurements were done in the temperature 35 °C. CPMG experiments were done and values of magnetization vector magnitude were recorded as a function of time for echo trains. *T*_2_ distributions were the final result of inversion made using BRD algorithm [36] realized in Oil and Gas Institute. After echo-fit signal amplitude was presented as a function of TC (time constant *T*_2_) in the range of 10–10,000,000 μs all results were related to calibrated standard.

**Fig. 10:**
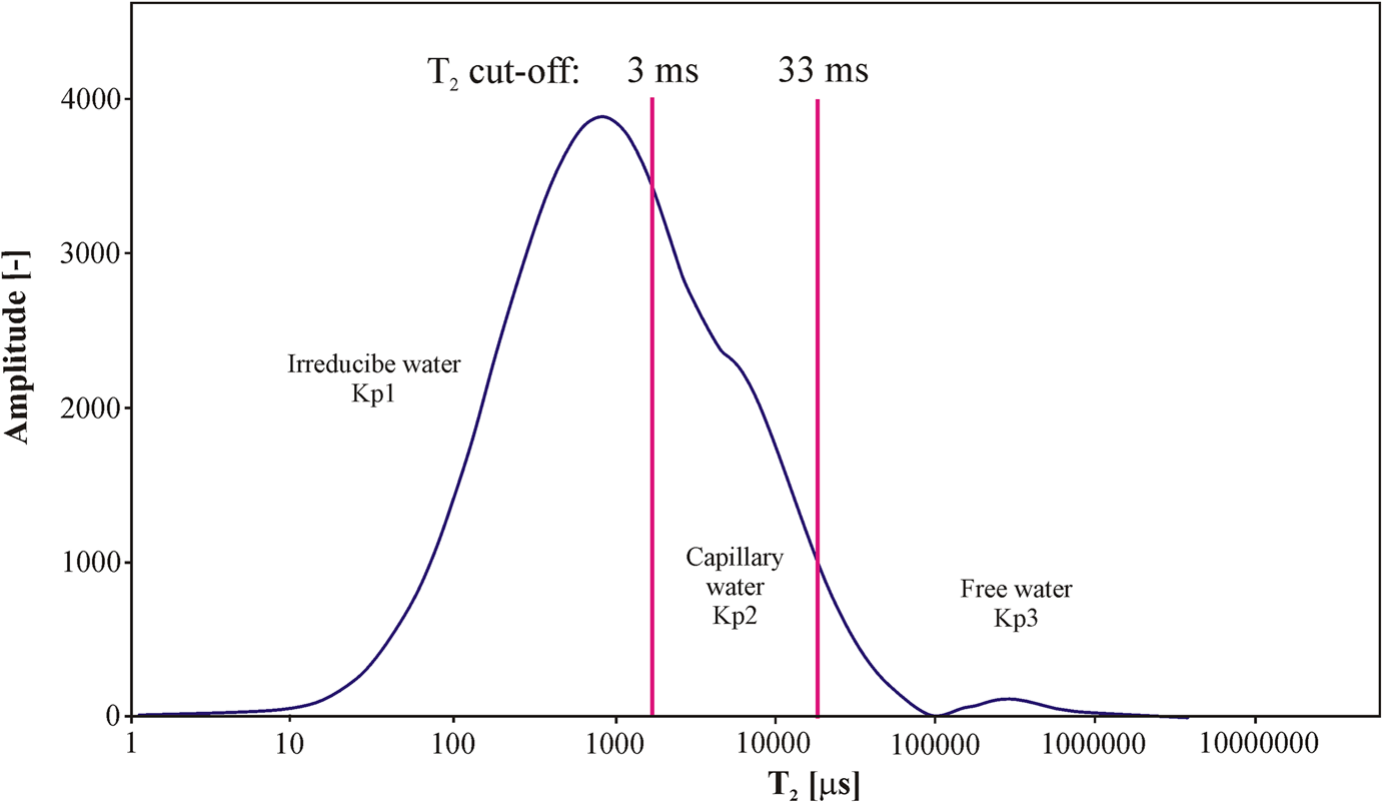
Standard presentation of NMR laboratory experiment results, M-1 well, sample 9831

NMR laboratory experiments provided the basic values, i.e. volume of irreducible water closed in clay minerals (Kp1), volume of capillary water bounded in small diameter pores (Kp2) and volume of free water which can be produced from rock formation (Kp3) (Fig. 10). In the next step Kp1, Kp2 and Kp3 were the start points to calculate the detailed information about porosity (total and effective) and irreducible water saturation [37]. The basis for the division of *T*_2_ distribution into three parts (Kp1, Kp2 and Kp3) is the adoption of proper cut-offs. They are determined as a result of combining outcomes of laboratory experiments with samples of variable saturation and NMR results obtained on the same samples. In this research we used typical cut-offs for clastic formations equal to 3 and 33 ms [38].

**Fig. 11:**
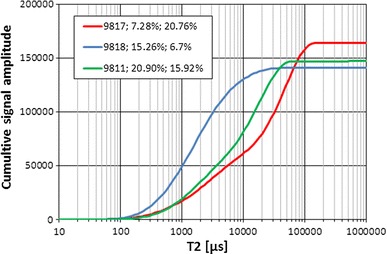
Cumulative *T*_2_ signals, curve parameter—*Φ*ef mp and Kp nmr ef, respectively

NMR curves presented in Figs. 11 (cumulative signals) and 12 (*T*_2_ distributions) are similar to the MIP plots–cumulative volume of mercury (Fig. 5) and incremental volume of mercury (Fig. 6), respectively. Results for the same core plugs were shown to enable the comparison of the courses of curves, their shapes and positions of the characteristic points. This primary similarity reflecting the physical processes of mercury intrusion into porous space and relaxation of hydrogen nuclei in volume of pores and on the surface of them was the basis for combining the specific parameters of both methods, i.e. volume of mercury, Bv all, Swanson coordinates, median pore diameter and Kp nmr, Kp nmr ef, NMR mean logarithmic *T*_2ML_, NMR average amplitude (Av_amp) etc. used in the comprehensive interpretation.

**Fig. 12:**
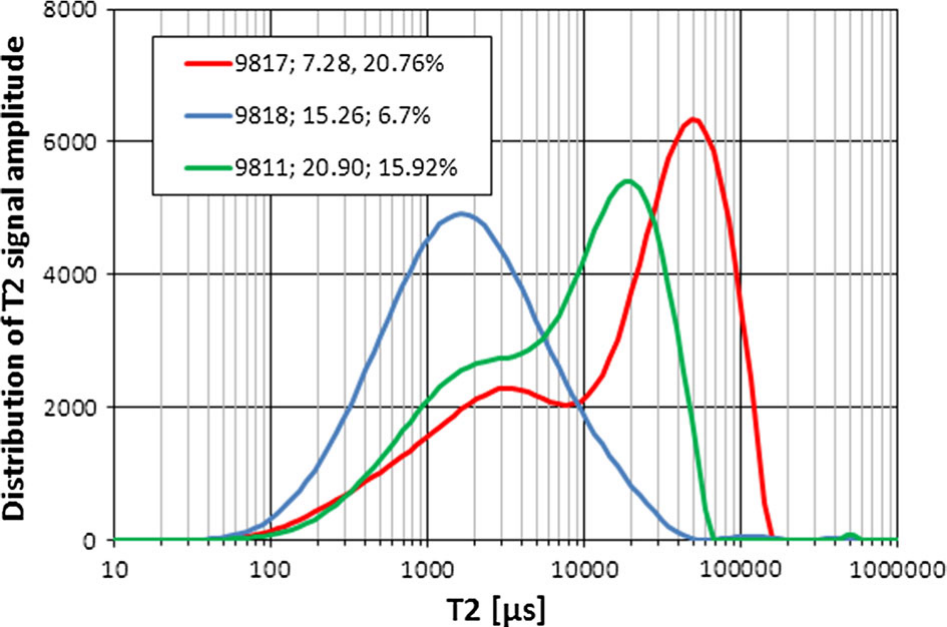
*T*_2_ signals, curve parameter—*Φ*ef mp and Kp nmr ef, respectively

NMR curves (Figs. 11, 12) were completed with information on effective porosity from MIP and effective porosity from NMR. Porosity from mercury injection porosimetry was before correction for boundary effect, so it was distinctly higher than Kp nmr ef excluding sample 9,817, for which higher Kp nmr ef was observed. Coupled approach to NMR signal plots made the analysis easier regarding values of cumulative signals (Fig. 11) and spike values and positions of them in *T*_2_ distributions (Fig. 12). The highest spikes (Fig. 12) were related to maximal Kp nmr and Kp nmr ef values and were located at the late *T*_2_ times, what was interpreted as contribution to NMR signal from large pores and fractures.

Following the division of MIP signals into three pore systems authors try to find similarity in NMR signals. Values labelled as *T*_2ML_ *–*NMR mean logarithmic *T*_2_ were calculated for full NMR signals (*TC* belonged to the range: 10–10,000,000 μs). Two additional values: *T*_2ML Kp1_, *T*_2ML Kp_ were calculated for two parts of NMR signals related to Kp1 (the first part) and Kp2 + Kp3 (the second part). Similar calculations were done for Av_amp, calculated for total NMR signal and Av_amp_1 and Av_amp_2–3 calculated for the first part of signal related to Kp1and the second part of the signal related to Kp2 and Kp3. Average amplitudes presented in figures below are normalized to maximal cumulative amplitude to make NMR signals of various plugs comparable. In next stage, factors described above were combined with other available parameters from standard laboratory measurements and MIP.

## Comprehensive Interpretation of Laboratory Data

The goal of the comprehensive interpretation was to build relationships between parameters and factors determined from rock sample laboratory investigations facilitating determination of one group of parameters on the basis of the other. The main purpose was to obtain parameters difficult to be parameterized to characterize the pore space and determine fluid flow ability in the Miocene sandstone-mudstone-claystone formation. All available parameters were engaged in the analysis covering wide range of aspects important in conventional reservoir characterization. Some of the presented relationships can be extended into unconventional reservoirs.

**Fig. 13:**
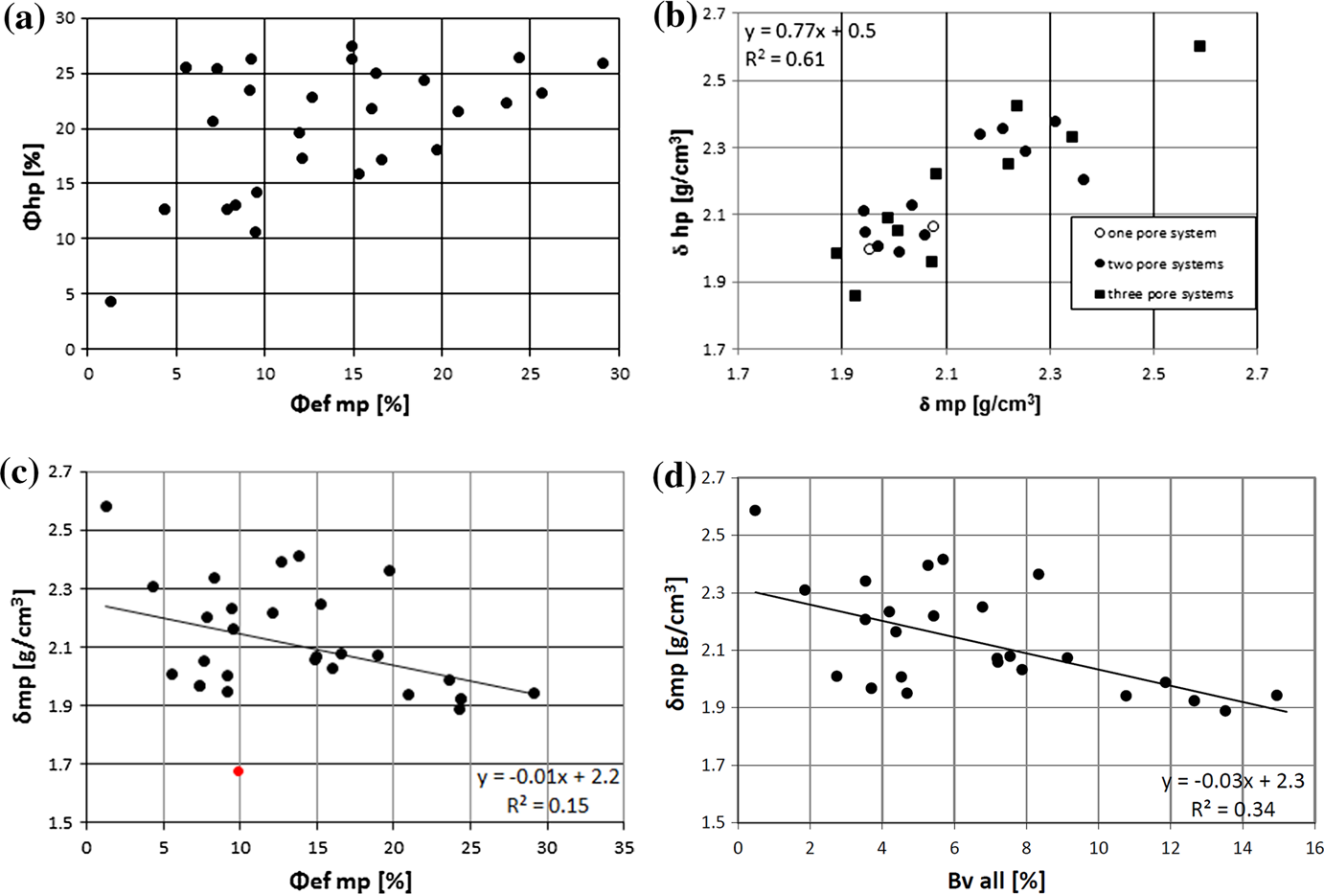
**a** Comparison of porosity values from HP, *Φ*_hp_, vs. MIP porosity,*Φ*ef mp. **b** Bulk density from helium pycnometer, *δ*_hp_, vs. *δ*_mp_ mercury porosimetry. **c** Bulk density from MIP, *δ*_mp_, vs. effective porosity from MIP, *Φ*ef m; one outstanding point marked in *red*. **d** Bulk density from MIP, *δ*_mp_, vs. effective porosity calculated as a sum of mercury volume injected in the pore system, Bv all (color figure online)

Comparison of the total porosity from helium pycnometer (HP) (*Φ*_hp_) and mercury injection porosimetry (MIP) (*Φ*_mp_) showed a great dispersion of data (Fig. 13a). In all samples HP porosity was higher than MIP porosity. Bulk density from HP (*Φ*_hp_) and bulk density from MIP (*Φ*_mp_) also presented dispersion but not so significant (Fig. 13b). In the MIP results three pore systems (SP1 + SP2 + SP3) were identified or two systems (SP1 + SP2) were distinguished or only one system (SP1) was visible. Data representing three groups were marked with different symbols (Fig. 13b). All data presented similar course: no differentiation according to system division was observed. Dispersion was related to properties of rocks and the accuracy of measurements.

Relationship between bulk density vs. porosity from MIP (Fig. 13c) showed higher dispersion than the plot of MIP bulk density vs. Bv all (Fig. 13d). Removing of one outstanding point (9.89, 1.68) improved correlation. Observed immediate change in the correlation showed that data dispersion caused lack of stability in considered relationships, mainly due to many petrophysical factors influencing results of laboratory experiments. On the basis of plots in Fig. 13 authors concluded that more reliable results were obtained from computer-processed MIP outcomes (Bv) and from HP data than raw porosimetry measurements.

**Fig. 14:**
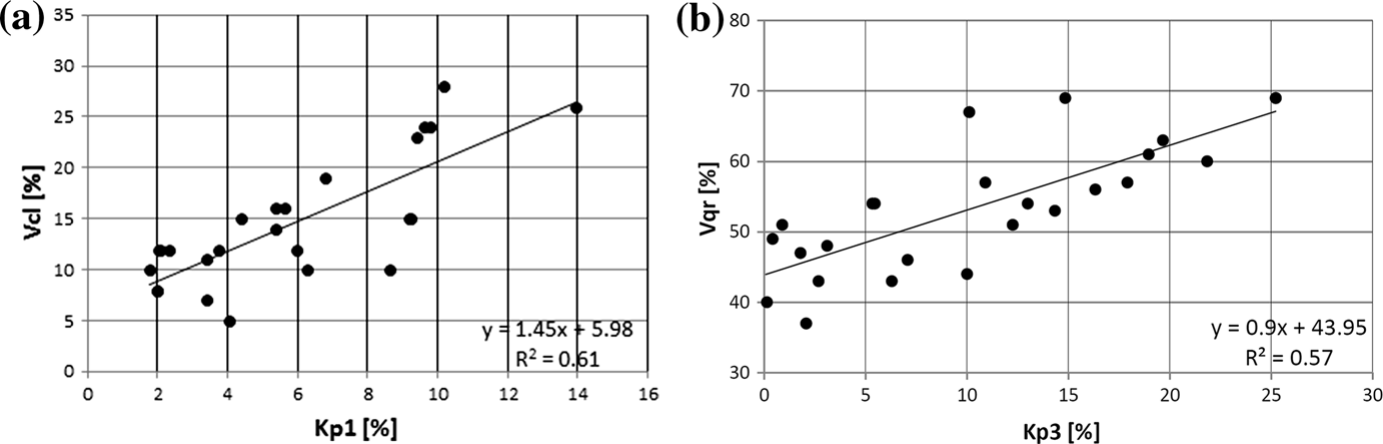
**a** Volume of clay minerals, Vcl, vs. volume of clay bound water, Kp1. **b** Volume of quartz, Vqr, vs. volume of movable water, Kp3

Similar relationships were tested between quantities determined from NMR experiments. Total porosity from NMR, Kp nmr, and HP porosity, *Φ*_hp_, were well correlated. High values of total porosity from NMR and low values of intruded mercury volume were observed in shaly formation. Results of the XRD analysis were the source information about shaliness (defined as the sum of clay minerals) of rock samples. The information was not highly credible due to the limited number of data but correlation between sum of clay minerals (Vcl) and Kp1 (Fig. 14a) confirmed influence of shaliness on the examined relationships. Also, presence of clay minerals (shaliness) in the Sarmatian rock formation explained high volume of irreducible water. Volume of quartz, Vqr, (from XRD analysis) mainly related to sandy laminas, corresponded to volume of movable water (Fig. 14b).

Quantities determined by NMR laboratory experiments were correlated with MIP outcomes to generate equations which could be used in prediction of difficult to be measured rock parameters. In the next figures two plots are presented. One is related to factors calculated for full NMR signal, the second one was calculated for the second part of *T*_2_ distribution (related to Kp2 + Kp3). Differences between two plots and differences in the coordinates of data on the vertical and horizontal axis reflected variability of NMR signals (Figs. 11, 12).

**Fig. 15:**
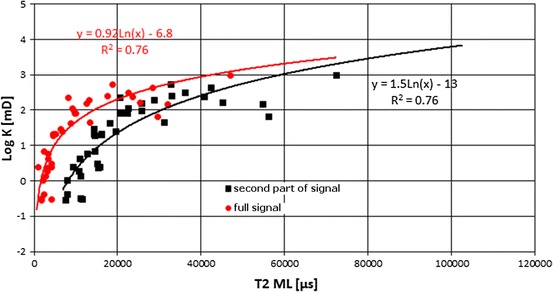
Logarithm of permeability, log *K*, vs. *T*_2_ logarithmic mean, *T*_2ML_; in *red*—relationship for full *T*_2_ distribution, in *black*—relationship for the second part of *T*_2_ distribution, *T*_2_ > 3 ms (color figure online)

*T*_2_ logarithmic mean calculated for the total signal (*T*_2ML_) was correlated with logarithm of permeability (log *K*) (Fig. 15). Increase of permeability caused increase of *T*_2ML_. High values of *T*_2ML_ mean late position of maximum on the *T*_2_ distribution, what is related to high permeability.

**Fig. 16:**
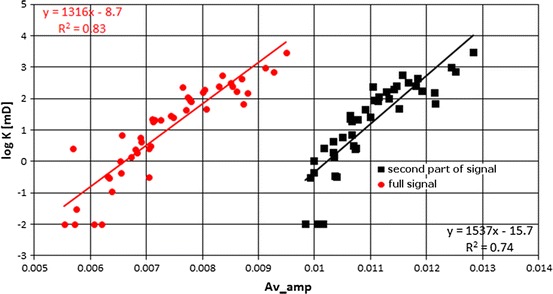
Logarithm of permeability, log *K*, vs. averaged amplitude Av_amp; in *red*—relationship for full *T*_2_ distribution, in *black*—relationship for the second part of *T*_2_ distribution, *T*_2_ > 3 ms (color figure online)

Relationship between logarithm of permeability and averaged amplitude was also considered (Fig. 16). Two plots are presented in Fig. 16. One was the result of averaging of the amplitude in full signal and was shifted to smaller *T*_2_ and revealed smaller amplitudes. The second was presented for the second part of *T*_2_ distribution, related to pores and fractures responsible for higher permeability. Authors followed the assumption that small pores in shaly part of the Miocene formation mostly influencing the first part of *T*_2_ distribution (below 3 ms) did not take part in fluid flow.

**Fig. 17:**
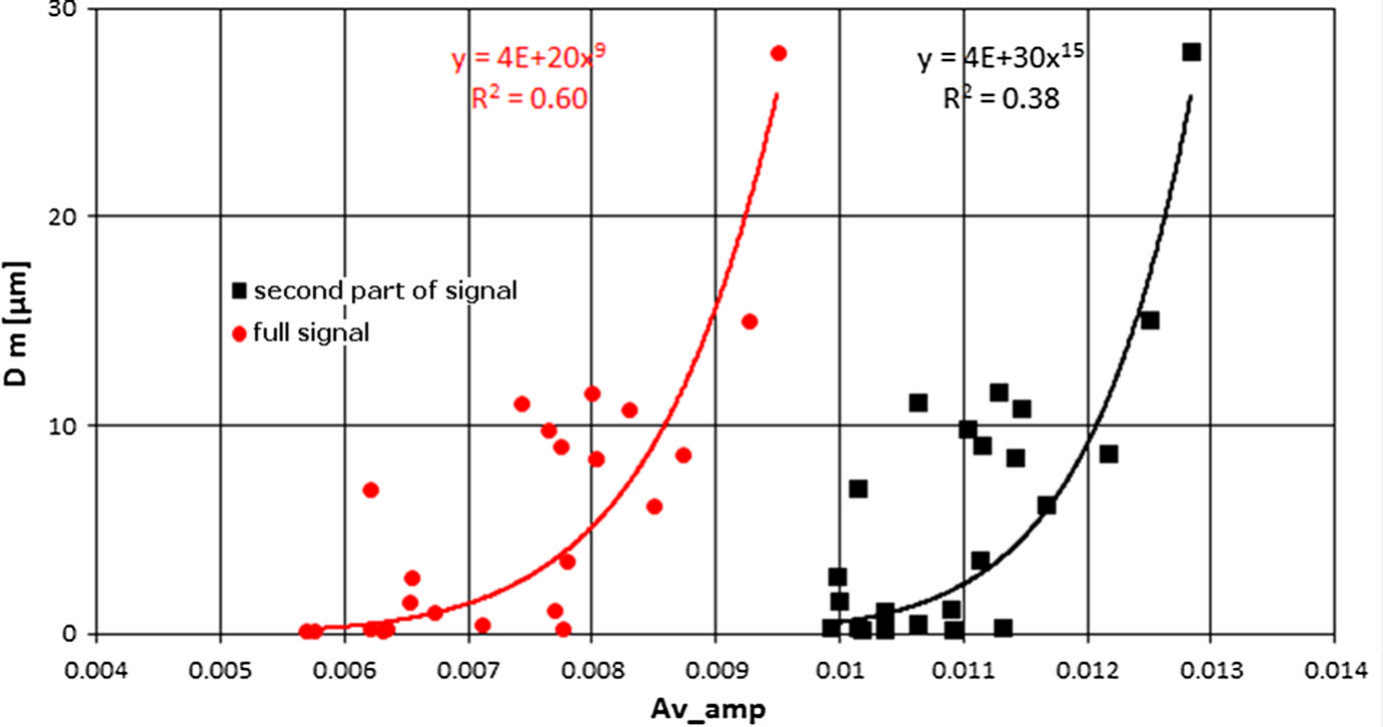
Median pore diameter, *D* *m*, vs. average amplitude, Av_amp; in *red*—relationship for full *T*_2_ distribution, in *black*—relationship for the second part of *T*_2_ distribution, *T*_2_ > 3 ms (color figure online)

Similar relationships were built for median pore diameter calculated on the basis of pore volume occupied by mercury vs. average amplitude (Fig. 17). Both plots determined from *T*_2_ distributions are related to reservoir properties, i.e. porosity (higher porosity–higher amplitude) and permeability (higher permeability–later position of maximum on time axis) defining fluid flow.

**Fig. 18:**
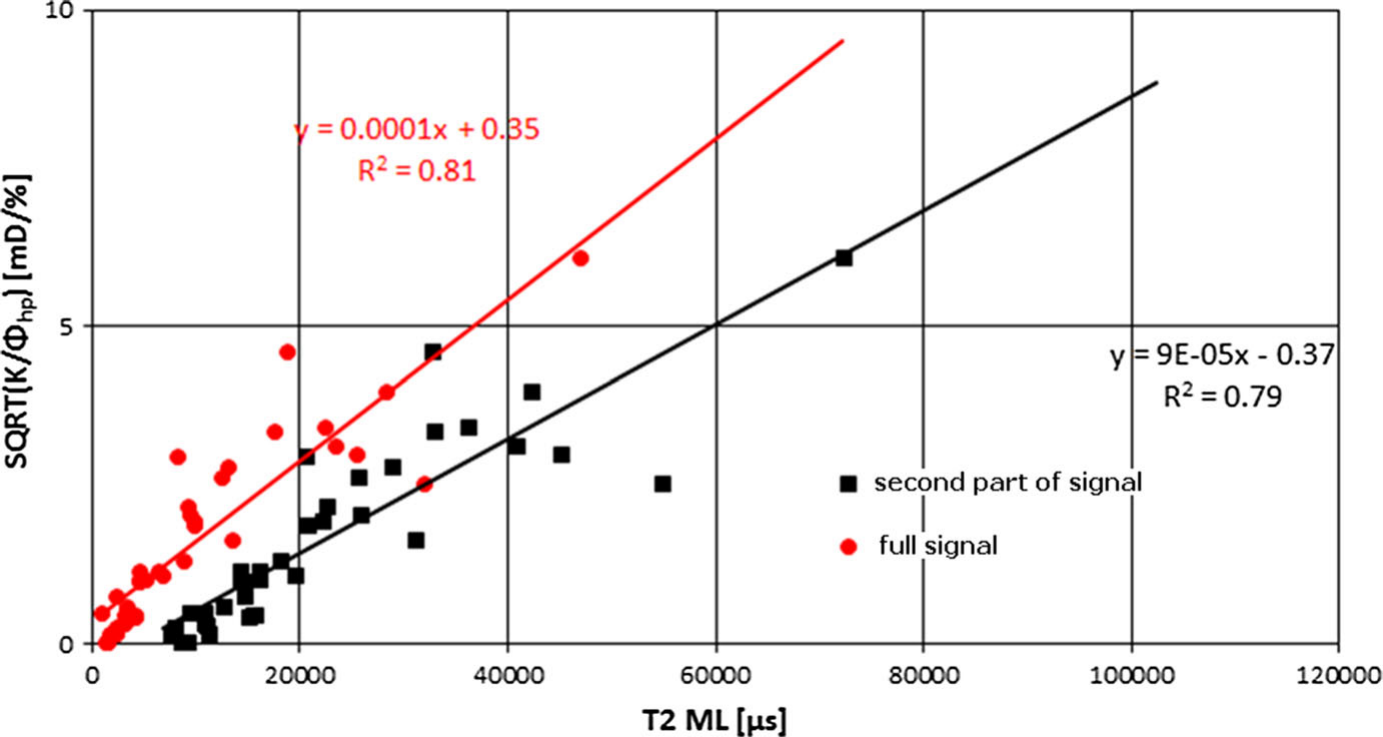
Relationship between SQRT (*K/Φ*_hp_) vs. *T*_2_ logarithmic mean, *T*_2ML_

Relationships between specific reservoir factor SQRT (*K/Φ*_hp_) which reflected the coupling between permeability and porosity and fluid-flow ability in porous formation vs. *T*_2_ logarithmic mean is presented in Fig. 18. Higher values of specific factor calculated for the full range of signal than for the second part of signal was related to the amplitude of the NMR signal closely combined with total porosity. *T*_2ML_ calculated in the second part of signal (Kp2 + Kp3 area) was influenced by maxima sited in the part of *T*_2_ distribution where *T*_2_ > 3 ms. Determination coefficients are similar, so both relationships can be used for permeability calculation.

**Fig. 19:**
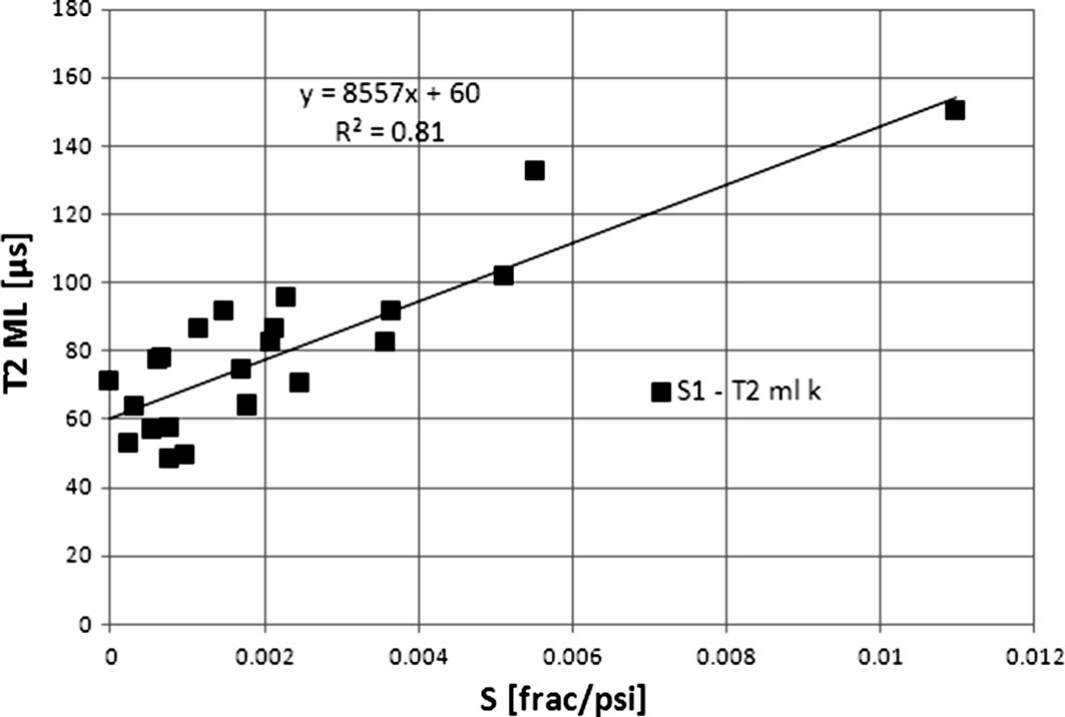
*T*_2_ logarithmic mean, *T*_2ML_, vs. Swanson parameter, *S*

**Fig. 20:**
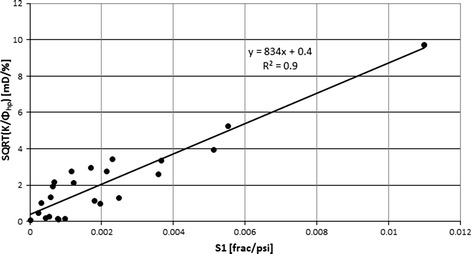
Specific factor, SQRT (*K/Φ*_hp_), vs. Swanson parameter in SP1 pore system, *S*1

The assumption that fluid flow takes place only in the connected pores of diameters confirmed by MIP was followed and correlation between Swanson parameter, *S*1, calculated for the SP1 system of pore space vs. *T*_2_ logarithmic mean, *T*_2ML Kp_ calculated for the second part of *T*_2_ distribution was presented (Fig. 19). Swanson parameters calculated for pore systems identified in rock sample (Fig. 7), related to Thomeer hyperbolas fitted to the MIP signals characterized the ability of fluid flow in the separated systems of pores. High correlation presented in Fig. 19 between *S*1 and *T*_2ML Kp_ showed that the SP1 pore system identified in MIP and the second part of NMR signal were crucial for fluid flow in the discussed rock formation. Swanson parameter combining pressure and volume of mercury intruded into pore space is also closely related to specific factor SQRT (*K/Φ*_hp_) defining the ability of fluid flow (Fig. 20).

## Conclusions

Comprehensive interpretation of the laboratory results from various methods, used for determination of reservoir properties of rocks, turned to be an effective way to improve the results obtained with individual methods. Porosity, being crucial reservoir property (together with permeability), was determined from helium pycnometer, HP, measurements, mercury injection porosimetry, MIP, and NMR experiments. All methods were based on different physical phenomena so, the results were mutually complementary.

Computer processing of laboratory outcomes provided new way for obtaining additional information apart from traditional aspects discussed in analysis of laboratory outcomes. Excluding part of porosity from MIP related to boundary effect provided more reliable values, correlating better with other results (HP and NMR). Division of pore space into independent pore systems on the basis of mercury injection porosimetry enabled construction of additional relationships between different parameters making the comprehensive interpretation easier and more precise.

Defining new parameters showed the way for improving the available data. Combination of single properties, i.e. porosity and permeability (calculating square root of permeability to porosity ratio) revealed new insight into known properties presenting hydraulic abilities in the discussed case.

Determination of the relationships between groups of quantities describing pore space of rock formation was presented as the basis for prediction and for relationships extrapolation into interesting areas. MIP results and NMR outcomes were combined to determine the relationships facilitating the reservoir characterization.

Limited number of coherent credible data was the main reason that confidence intervals were not calculated for presented relationships, enabling reliable prediction of properties which are difficult to be determined (permeability, pore diameter) on the basis of easy measured quantity (porosity). Disposing the complete data set from MIP and NMR experiments completed with standard pycnometer porosity and bulk density measurements together with XRD analysis for mineral composition and credible permeability determination provided effective reliable tool for complete reservoir properties analysis.

Investigated plots between various parameters and factors selected to the comprehensive analysis showed that data are dispersed and relationships are not stable, mainly due to many petrophysical factors influencing results of lab experiments. Part of this instability can be explained by great variability of lithological and petrophysical features of rocks and partially by not sufficient accuracy of measurements. The last part is related to subjective approach of interpreter in semiautomatic processing of MIP.

## List of Symbols

MIP: Mercury injection porosimetry
HP: Helium pycnometer
NMR: Nuclear magnetic resonance
*S* Hg [ml/g]: Cumulative volume of mercury
*S* Hg inc [ml/g]: Incremental volume of mercury
*A* [m^2^/g]: Cumulative pore area
*A* inc [m^2^/g]: Incremental pore area
*D* [μm]: Pore diameter
*Dm* [μm]: Median pore diameter
*Φ*ef mp [%]: Effective porosity from MIP
SP*i, i* = 1, 2, 3: Selected pore system obtained from processing of MIP results using Thomeer hyperbolas
Bv_*i*_, for *i* = 1, 2, 3 [%]: Bulk volume occupied by mercury in selected pore system (SP1, SP2 or SP3), effective porosity of selected pore system
Pd_*i*_*, for i* = 1, 2, 3 [psi]: Pressure at which mercury started to intrude into selected pore system (SP1, SP2 or SP3), threshold pressure or extrapolated displacement pressure
Bv all [%]: Effective porosity calculated as a sum of mercury volume injected in the determined porous system
*S*_*i*_*, i* = 1, 2, 3: Swanson parameter in selected pore system (SP1, SP2 or SP3)
Spc [psi]: Swanson parameter coordinate referring to *Y* axis (pressure) on log–log graph of bulk volume occupied by mercury and pressure from MIP
Sbv [%]: Swanson parameter coordinate referring to *X* axis (volume of intruded mercury) on log–log plot of bulk volume occupied by mercury and pressure from MIP
*P*c [psi]: Pore pressure
Kp nmr [%]: Total porosity from NMR measurement
Kp nmr ef [%]: Effective porosity from NMR measurement
Swir [%]: Irreducible water saturation from NMR measurement
Kp1 [%]: Volume of irreducible water
Kp2 [%]: Volume of capillary-bound water
Kp3 [%]: Volume of moveable water; free fluid index (FFI); dynamic porosity
Vcl [%]: Volume of clay minerals from XRD measurement; shaliness
Vqr [%]: Volume of quartz from XRD measurement
*T*_2ML_: *T*_2_ logarithmic mean from NMR measurement
AS_*i*_: NMR signal amplitude
*T*_2ML Kp1_: *T*_2_ logarithmic mean from NMR measurement calculated for the part of NMR signal related to Kp1
*T*_2ML Kp_: *T*_2_ logarithmic mean from NMR measurement calculated for the part of NMR signal related to the Kp2 + Kp3
Av_amp: NMR average amplitude
Av_amp_1: NMR average amplitude calculated for part of signal related to Kp1
Av_amp_2–3: NMR average amplitude calculated for parts of signal related to Kp2 and Kp3
*Φ*_hp_ [%]: Effective porosity from HP
*δ*_hp_ [g/cm^3^]: Bulk density from HP
*δ*_mp_ [g/cm^3^]: Bulk density from MIP
*K* [mD]: Absolute permeability
SQRT (*K/Φ*_hp_): Square root of permeability to porosity from HP ratio
*R*^2^: Determination coefficient
